# Training needs assessment of hospital CEOs in a developing country: the example of Iran

**DOI:** 10.1186/s12909-023-04463-2

**Published:** 2023-08-24

**Authors:** Seyede-Elahe Hosseini, Mehdi Jafari, Ali Nemati, Keyvan Rahmani, Payam Mahmoudian, Masoud Ferdosi

**Affiliations:** 1 Health Management and Economics Research Center, School of Management and Medical Information Sciences, Isfahan University of Medical Sciences, Isfahan, Iran; 2 Department of health Services Management, School of Health Management and Information Sciences, Iran University of Medical Sciences, Tehran, Iran; 3 Department of Health Service Management, School of Management and Medical Information Sciences, Iran University of Medical Sciences, Tehran, Iran; 4 Health Management Development Institute, Ministry of Health and Medical Education, Tehran, Iran; 5 Health Management and Economics Research Center, School of Management and Medical Information Sciences, Iran University of Medical Sciences, Tehran, Iran; 6 Health Management and Economics Research Center, Department of Health Services Management, School of Management and Medical Information Sciences, Isfahan University of Medical Sciences, Isfahan, Iran

**Keywords:** Needs assessment, Competency-based education, Chief executive officers, Hospital

## Abstract

**Background:**

It is essential to identify the necessary competencies of hospital CEOs in order to improve the quality and efficiency of services they provide. Expert leadership skills and competencies can have a significant impact on the success of an organization, benefiting both patients and staff. This study aimed to assess the competencies and training needs of hospital CEOs in Iran public hospitals.

**Methods:**

We conducted this cross-sectional analytical study through a self-assessment questionnaire, which was a web-based platform developed by the WHO country office in Iran, between July 2018 and September 2018. The questionnaire was completed by 180 hospital CEOs and included a core set of 81 items based on Assessing the Competency of Hospital CEO. These items were categorized into five superordinate categories: leadership, personality and quality of individual behavior, knowledge and business skills, social responsibility, and healthcare environment. In addition, we conducted focus groups with 30 hospital CEOs, supervisor assessments with 10 hospital managers, and interviews with 10 supervisors.

**Results:**

Of the 180 questionnaires distributed, 78% were returned, and most respondents were medical specialists. The need for leadership competencies such as individual behavior skills and change management received the highest priority. Most respondents required training in management skills, including financial management, governance, strategic thinking, quality improvement, and disaster management.

**Conclusion:**

Providing needs-based education is crucial, especially in developing countries. In this study, leadership and strategic thinking were found to be the most needed competencies among hospital CEOs in Iran. These findings serve as reference points for developing countries with similar backgrounds and healthcare environments as Iran.

**Supplementary Information:**

The online version contains supplementary material available at 10.1186/s12909-023-04463-2.

## Background

The healthcare sector is facing numerous challenges today, such as increasing demands for medical services from the community and decreasing budgets. As a result, the roles of healthcare managers are becoming increasingly complex and challenging. It is essential for healthcare managers to make informed decisions that balance financial performance with the need for high-quality patient care [1].

Hospitals are vital institutions for providing healthcare services. The World Health Organization recognizes the crucial role that hospitals play in achieving health system goals [2]. To enhance the quality of management in hospitals, it is crucial to ensure that they are staffed with highly skilled and competent managers [3]. On the other hand, the growth and progress of nations and organizations are closely linked to creativity and innovation in the development of human resources [4]. Because Human resources are considered as the main capital and one of the factors to achieve the goals of organizations [5]. management literature confirms that managerial competence plays a crucial role in supporting high performance organizations and systems [6]. Professional competencies have garnered much attention and concern from managers and nurses who work in both management and patient care. As a result, identifying and developing these competencies has become a challenge and a focal point of interest for all stakeholders involved, particularly in the hospital setting [7]. Leadership is widely acknowledged as one of the most critical factors that can significantly impact an organization’s performance and overall level of excellence [8]. Managerial competence can be described as a combination of knowledge, skills, behaviors, abilities, and attitudes that contribute to the overall effectiveness of a manager [9]. Competent managers typically exhibit specific competencies, which encompass knowledge, behavior, skills, attitudes, and values that are related to job performance [6]. The identification of competencies necessary for successful leadership in a healthcare organization is dependent on the specific clinical and administrative leadership roles.

In Iran, the Ministry of Health and Medical Education (MOHME) is responsible for providing healthcare services. This includes both public and private providers, including for-profit and non-profit organizations, as well as charity-based healthcare service providers. Currently, there are a total of 956 hospitals in Iran, with 125,265 beds available for patients. Out of these hospitals, 592 are affiliated with universities or government-run hospitals (62%), while 73 are associated with the Social Security organization (8%). Additionally, there are 166 private hospitals (17%) and 125 other hospitals (13%), which are either military-based, owned by banks, charities, or other entities. In Iran, the healthcare system is composed of three levels: specialty and super-specialty healthcare services, district health networks (district public hospitals, and specialized polyclinics), and primary care services (community health centers and health houses). The majority of major hospitals in Iran are affiliated with universities or government-run hospitals. However, other entities such as the Social Security organization, private sector organizations, and other entities including the military, banks, charities, etc., also manage hospitals. Table 1 presents the distribution of hospitals according to ownership. Hospitals are among the sectors where the importance of managerial competencies is most prominent [10]. It is essential to determine the necessary competencies of hospital CEOs to improve the efficiency and quality of healthcare services. Possessing expert leadership skills and competencies can also significantly impact an organization’s success, benefiting patients, staff members, and society as a whole [11]. Because Hospital CEOs hold an important role in promoting positive patient experiences [12].

**Table 1:** Hospital Sector Information in Iran

	# hospital	% of hospital	# Beds	% of Beds
Medical University (Public)	600	61.92	85,455	68.27
Private	166	17.36	16,232	12.97
SS0	73	7.64	10,644	8.50
Charities	36	3.77	3997	3.2
Others	89	0.09	8836	7.05
Total	956	100	125,265	100

In a 2023 study conducted in Kenya by Lekaldero et al., it was shown that management competence has a positive impact on increasing financial sustainability. As a result, the authors recommend that community conservation management must improve aspects related to management competence and ensure that their managers possess the necessary attitude, skills, and knowledge [13]. Another study examined the relationship between the competence of managers and the efficiency of teaching hospitals at Tehran University of Medical Sciences. The study found a significant relationship between leadership and planning competencies with bed occupancy rates [14].

Although there are many studies on managerial competencies in the field of health, there is no common consensus on the exact competencies required [15]. The purpose of this study is to identify a set of essential competencies for hospital CEOs in Iran. The study specifically focuses on hospital CEOs because, within the public sector in Iran, they are responsible for directing and coordinating various activities, including educational, administrative, research, and therapeutic initiatives. Furthermore, hospital CEOs are accountable for overseeing financial and administrative affairs related to the hospital.

## Methods

### Study design

The study employed a mixed-methods approach consisting of two stages carried out between July 2018 and September 2018. The first stage involved a 360-degree evaluation, which included self-evaluation using a questionnaire, evaluations from supervisors, peers, and subordinates. In the second stage, focus group discussions (FGD) were conducted with hospital CEOs to validate the key tasks for them and identify the essential competencies required to perform effectively. Since the study aimed to serve as a basis for developing a capacity-building program for hospital CEOs in the country, it was crucial to design the second stage as a qualitative study that aligned with the content of the needs assessment obtained in the first stage and considered the challenges of the country and stakeholders’ views. Therefore, hospital CEOs with experience in the current challenges of Iran’s health system were selected to participate in this stage to develop a practical training package based on the findings.

### Instrument

This study used the WHO regional office questionnaire and survey monkey, web-based platform developed by the WHO country office in Iran and health managers development institute (HMDI). The questionnaire is derived from the IHF global competency directory and consisted of five domains, including leadership (with two subdomains), personality and quality of individual behavior (with three subdomains), knowledge and business skills (with 28 subdomains), social responsibility (with five subdomains), and healthcare environment (with five subdomains). The questionnaire utilized a 5-point Likert scale ranging from 1 (not relevant) to 5 (high need) to assess the characteristics of each domain. The questionnaire was translated and validated by a group of experts and hospital managers, confirming its content validity. Additionally, its reliability was confirmed through Cronbach’s alpha coefficient (r = 0.97). The questionnaire also included a section for collecting biographical and occupational information.

### Sampling methods, data collection and analysis

In the first phase of the study, a census sampling method was employed to gather data from all teaching hospitals in the country, which comprised a total of 267 hospitals. Of these hospitals, 234 were affiliated with universities of medical sciences and constituted hospitals that were under the supervision of the country’s universities of medical sciences and had educational activities. The questionnaire was administered to the CEOs of these hospitals, resulting in the distribution of 234 questionnaires via mail to all universities of medical science. Between July 2018 and September 2018, a total of 180 hospital CEOs completed and returned the questionnaire, representing a response rate of approximately 77%. Finally, data obtained from Survey Monkey software were imported into SPSS version 21 for statistical analysis.

During the second phase, we conducted a focus group with key informants. This group included 30 hospital CEOs, 10 hospital managers and supervisors, and 10 deputy affairs of medical sciences universities. We selected these individuals based on their work experience, management skills, the size, type, and specialty of their hospitals.

The first step in our study was to identify the main tasks that health managers typically spend most of their working hours on. We then determined the most important competencies, including knowledge, skills, and attitudes, required for each task and established corresponding criteria for effective performance. Participants were surveyed to identify essential competencies needed for effective job performance.

During focus group discussions, we focused on hospital management competencies and prioritized the most critical competencies required for hospital CEOs.

An 8-hour group discussion session on priority competencies for training for hospital CEOs was held. In this meeting, the results of the first stage, which was in the form of a 360 evaluation, were presented to the participants, and then the list was provided to them. Then a person as a coordinator led the meeting and presented the purpose of holding the meeting to modify and make the priority list real.

In the first round, priorities were discussed and some competencies were added and subtracted. In the second round, prioritization was done again with the cooperation of all participants, and the output of the priorities counted from both stages is presented in Table 2.

**Table 2:** Hospital CEOs core competencies

	Competency: knowledge, Skills, Attitude
1	leadership
2	Proper interaction with hospital Manager
3	System thinking
4	Participatory Management
5	Strategic thinking and planning
6	Familiarity to rules and regulations
7	Team work
8	Familiarity with the role and function of CEO
9	Problem solving skills
10	Disaster Management
11	Risk management and patient safety
12	Negotiation techniques
13	Professional Ethics
14	Prioritization
15	Improving quality and accreditation
16	Team building ability
17	Control and monitoring
18	Motivation
19	Communication
20	Decision Making Techniques
21	Ability to analyze the internal environment of the hospital
22	Human resource skills
23	Operational Planning
24	Familiarity with the role and function of the hospital
25	Change management
26	Time management
27	Accountability and interaction with the patient
28	Manage meetings
29	Hospital organization skills
30	Measuring and managing performance
31	Assessment and evaluation skills
31	Social Responsibility
32	Ability to analyze external environment (economic, political, social, cultural)
33	Responsiveness and interaction with the community
34	Active listening skills
35	Financial Management
36	Analysis and evaluation of performance indicators
37	creativity and innovation
38	Supply Chain Management
39	Understanding the organizational culture
40	Conflict Management
41	Stakeholder Recognition
42	Facility Management
43	Evidence-based management
44	Critical Thinking
45	Project Management
46	Marketing
47	General contracting

For content analysis, each item was discussed one by one, and the attendees reached a qualitative consensus on each item, and some topics were merged at this stage.

Finally, the list of required competencies were quantitatively re-prioritized.

## Results

In all, 234 questionnaires were distributed; the response rate was 78%. The participants were 155 male (86%) and 25 female (14%). 74% people aged between 35 and 50 years. The characteristics of participants in survey presented in Table 3. 88% of CEOs were physician. Also about 80% hospital CEOs have not formal training or management course.

**Table 3:** characteristic of participants

gender	Male	155 (86%)	Education	Specialist	64%
	Female	25 (14%)		MD	24%
Age	≤ 34	7%		PhD	2%
	35–50	74%		MSC	6%
	≥ 50	19%		BSC	4%
The years’ work on this position	≤ 5	70%	**Experience**	≤ 5	60%
	5–10	13%		5–10	23%
	≥ 10	17%		≥ 10	11%
				No Experience	6%
Hospital beds	≤ 50	12%	**Management course**	22%	
	50–100	28%	**Informal Education**	23%	
	100–300	48%			
	300–600	10%			
	600–1000	2%			

**Fig. 1:**
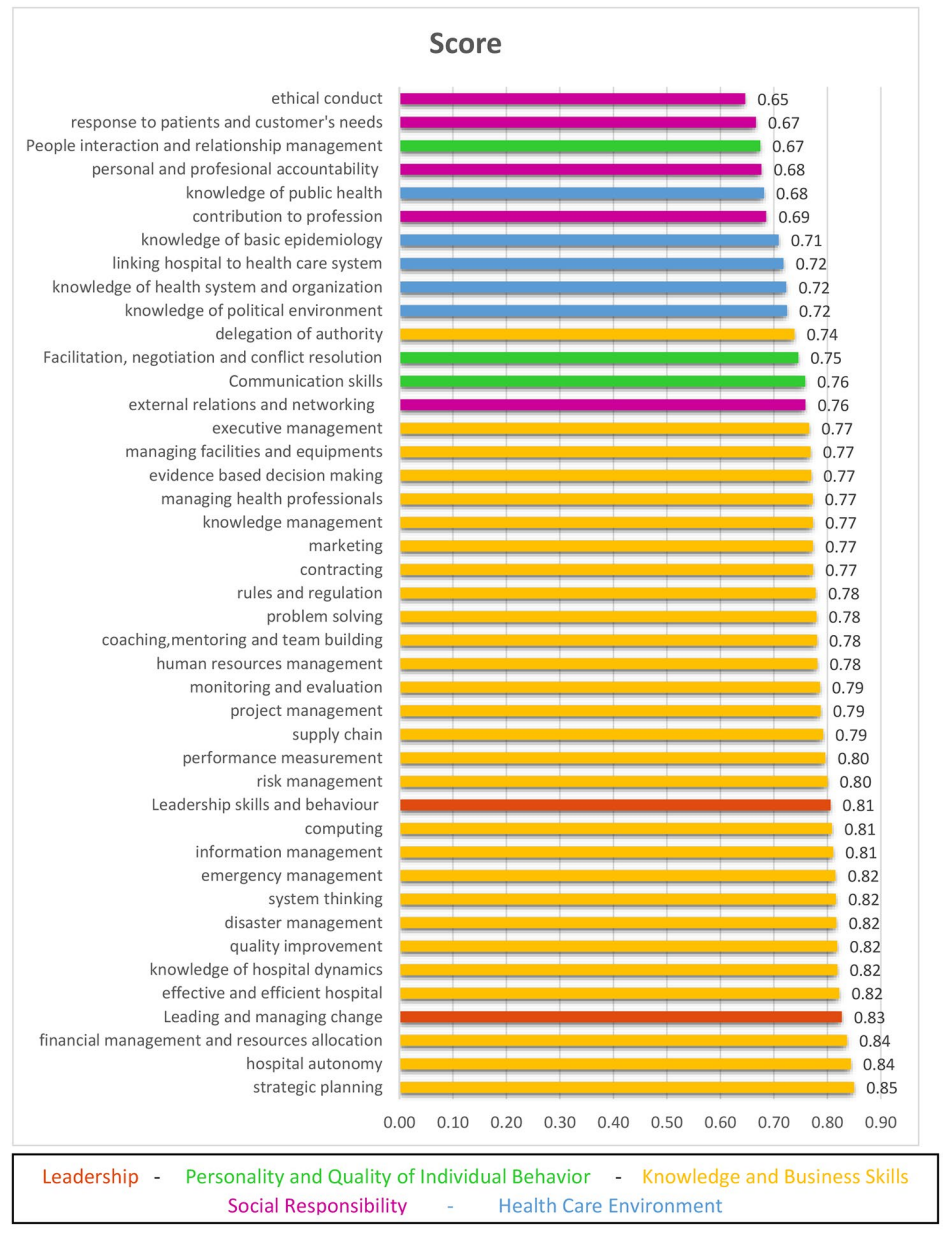
Score of subdomains

**Fig. 2:**
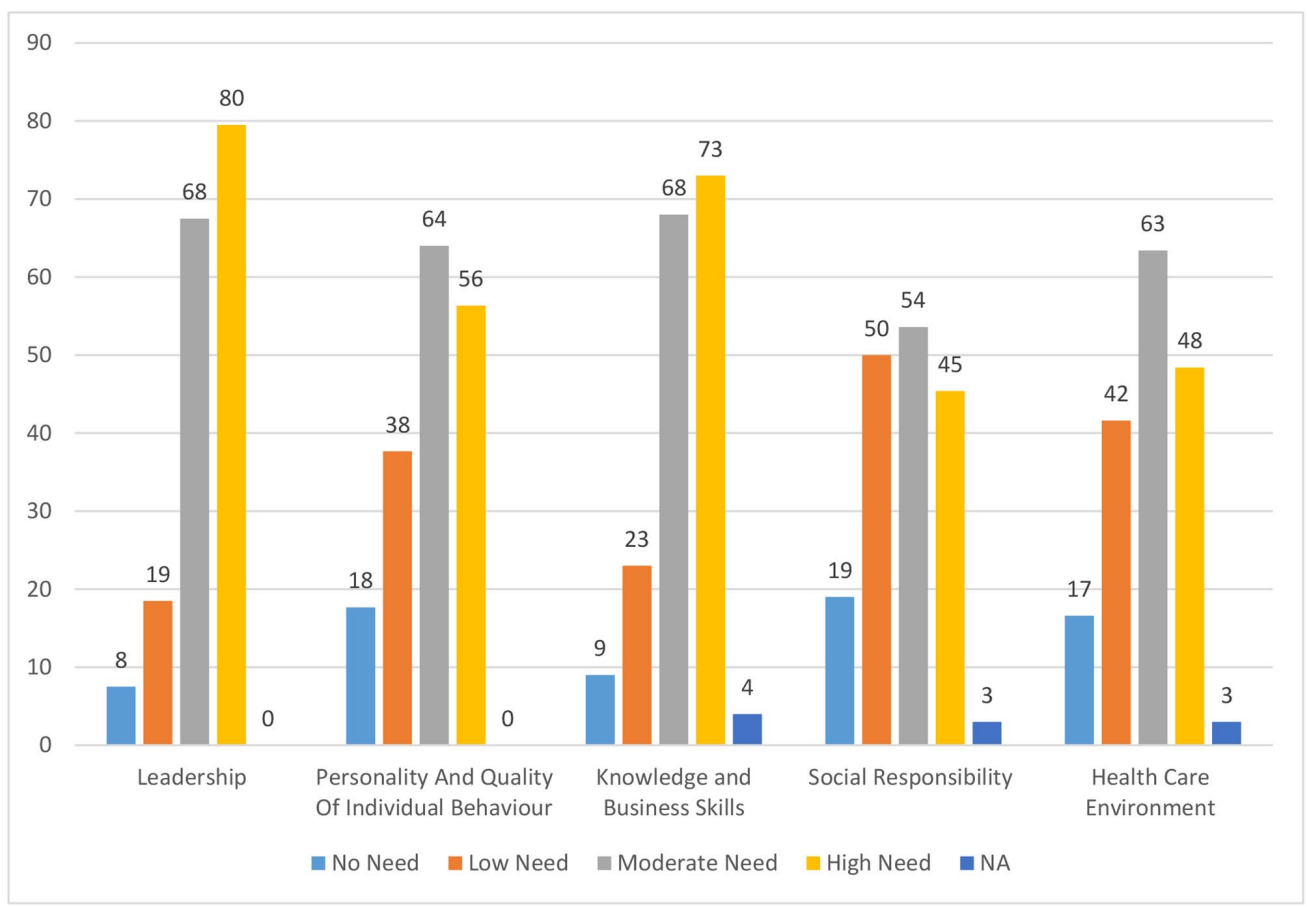
Needs expressed by the respondents in 5 dimentions

There is moderate and high need in two subdomains of the leadership category (Leadership skills and behavior / Leading and managing change). In the sub-domains of personality and the quality of individual behavior, there are communication skills and facilitation, negotiation and conflict resolution. In the category of business skills, there is a high need for strategic thinking, system thinking, hospital governance, knowledge of hospital dynamics, financial management and resource allocation, effective and efficient hospital, quality improvement, disaster management, emergency management and computing. In the subdomains of social responsibility, there is a high and moderate need for personal and professional accountability, participation in the profession, and response to patients. Finally, in the health care environment category, there are high and medium needs in all five subdomains (Fig. 1). We sorted the subdomains based on the highest to the lowest score (priority) and showed the subdomains of each category with a specific color. Also the measure of participants’ needs in 5 domains is specified in Fig. 2.

In addition to need assessment, core competencies were addressed in a focus group discussion and a framework of competencies required for hospital CEOs was proposed. According to the meeting with 30 hospital directors, 10 hospital manager and supervisors and 10 deputy affaires of University of medical science expressed the competencies for the hospital CEOs (Table 2). Because Findings of this assessment was used for developing and implementing a customized training course for hospital CEOs.

Base on this Results, the International hospital federation cooperating with WHO has designed Modules covering five domain for Hospital CEOs in Iran. And this training course was held in December 2018. The details of this program are given in Table 4.

**Table 4:** Leadership and management Program for hospital CEOs

Domain	Topics (Modules)
Know yourself and manage your self	Understand yourself
	Understanding leadership
	Managing and developing yourself as a leader
	Acting as an agent of change
Understand constraints and opportunities, interact with people	Understanding and managing organization culture
	Key external constraints and opportunities
	People management
	Trust building
	Team development
Understand the dynamic od decision making	Types of decisions
	Principles of decision making
	Orientation for decision- making
Prioritize make decisions and adopt decisions	Prioritizing and decision making
	Ethics in decision
	Adoption and diffusion a decision
Manage your resources, monitor your processes	Human resource Management
	Financial management
	Purchasing and procurement
	Equipment and facility management
	Information and knowledge management
	Processes and flow management
Manage performance, prepare your organization for future	Strategic Planning
	Project and change management
	Performance management
	Risk management
	Governance principle and practice
	Becoming a learning organization

## Discussion

This study identified the essential competencies for hospital CEOs to provide better services in our organization. According to the findings of this study, among the examined domains, the highest level of need was for the leadership domain, which includes Leadership skills and behavior and Leading and managing change. In Liang study the authors declared despite the diversity and range of tasks required of managers from different levels, there are common core competencies at all three levels. The results of the focus group and online survey led to the identification of six core competencies: leadership and change management; resource management; evidence-based decision making; knowledge of the health care environment and organization; Communication skills and relationship management [16]. In the present study, change management was one of the high-priority competencies, but the competencies related to the health care environment had relatively lower priority.

As mentioned in the findings, Knowledge and business skills were the most needed among the participants. In this regard in a systematic review by Kakemem et all, new competency model designed that includes the following seven core leadership and management competencies which is consistent with the results of the present study in knowledge and business skills, personality and the quality of individual behavior and also leadership domains. In another qualitative study conducted by Barati et al. regarding the competencies required by managers, managerial skills and financial awareness were the key issues identified [17]. In present study, among the management skills, financial management was one of the subjects with high priority. The results of the analysis about leadership and managerial competencies for public hospital managers in Vietnam also confirm financial management competencies [18].

As the results of the study show, there is a need to strengthen Knowledge and management skills, such as financial and resource management, hospital management, strategic thinking, quality improvement, disaster management, emergency management and computing. Crisis management competency is seen among the management skills with high priority. Since disaster preparedness plays an important role in hospital management [19], and following the major incidents and disasters in the country managers should plan in the disaster management [20]. So it is necessary for hospital CEOs to strengthen this competency. Also in Gaspard study the need for continuing professional education was rated the highest priority, followed by Computer skills, Disaster management and Health information management [21], which was consistent with our survey results.

In Pillari’s study, which was conducted among managers of public and private sector hospitals in South Africa and through a questionnaire, it also showed that the importance of competencies related to “people management”, “self-management” and then “strategic planning” was more important [22]. In our study, strategic planning and management gained the highest priority both in the quantitative stage and in the competencies obtained from the focus group stage.

In This study and it is in accordance with similar studies, 64% of hospital CEOs have limited proficiency in many identified competencies. This may be the result of that majority of hospital CEOs were clinicians having received limited management and leadership trainings. In addition, most of them have less than 5 years’ work experience in current position Therefore the result from the assessment is identifying training needs that mirror well the expected shortcomings.

The findings of this study support the competency-based educational approach for the training and preparation of current and future hospital CEOs. The main competencies identified can provide a very useful guide to identify competency gaps and management training needs and development of this category of managers. Because according to the researcher’s studies, no study has been done regarding the competencies of hospital CEOs in the country. In This study, the research community did not include all hospitals in the country. Because it was difficult to access other hospitals such as social security, private, etc. Also, we had limitations in conducting a comprehensive 360 evaluation, especially for the selection of subordinates. In total, the results of the study seem to be valid and generalizable at the level of hospitals in the country.

## Conclusion

Providing needs-based education is critical, especially in developing countries. In this study, leadership and strategic thinking was found to be the most required among hospital CEOs in Iran.

The results indicate a significant need for the continued development of hospital CEOs. It is the responsibility of administrators and those accountable for management education and training to identify managers who require further development and provide them with appropriate training that is contextually relevant in both design and delivery. Additionally, future studies should aim to create a framework for assessing, choosing, and appointing hospital CEOs based on their job competencies.

### Electronic supplementary material

Below is the link to the electronic supplementary material.

Supplementary Material 1

## Data Availability

The dataset supporting the conclusions of this article is included within the article as Additional file 1.

## Abbreviations

CEO: Chief Executive Officer
IHF: International Hospital Federation
