# Supplementary material for: Training needs assessment of hospital CEOs in a developing country: the example of Iran
Source: BMC Med Educ. 2023 Aug 24;23:602. doi: 10.1186/s12909-023-04463-2 (PMC10464028; doi:10.1186/s12909-023-04463-2)
Supplement: Supplementary file 1 — Supplementary Material 1 [file 12909_2023_4463_MOESM1_ESM.docx]

| Training Needs Assessment for Public Hospital Managers |  |
| --- | --- |
| INTRODUCTION |  |
| **Hospitals represent a key provider of health services in all countries. Hospital managers are to a large extent responsible for operationalizing the visions and objectives that policy-makers have for the health and well-being of a nation. Hospital managers need to possess a range of competencies (knowledge, skills, behaviors, and attitudes) which enable them to perform their managerial functions effectively and efficiently. To improve these competencies, hospital managers are the subject of continuous competency-based education and training.**  **It is important to determine training needs of senior hospital managers which assist in development of training programs in hospital care management. This survey serves this purpose.**  **The Hospital managers at level I (Chief Executive Officer-CEO/Hospital Director) and Level 2 (managers who report directly to CEO/Hospital Director) are requested to complete the Survey.**  **The completion of the questionnaire will take approximately 10-15 minutes of your time.**  **BEFORE RESPONDING TO THE SURVEY, PLEASE NOTE THE FOLLOWING:**   1. **Survey can be completed in several sessions** 2. **Responses per page are automatically saved** |  |

Training Needs Assessment for Public Hospital Managers

Demographic Characteristics of the Respondent

# Gender

Male Female

# Age (years)

< 35

35-50

>50

# Current Designation

CEO/Hospital Director

Hospital manager/Administrator Administrative manager

Other (please specify)

# Academic Degree

Bachelor Master PhD

Other (please specify)

# Formal Qualification

Medical/Health related Health care management

Commerce/General management More than 1 of above 3

Other (please specify)

# Years in Current Position

<5

5-10

>10

# Experience in Hospital Management (Duration in years)

<5

5-10

>10

# Formal training in Hospital Management

Yes No

If yes, please specify title, date, organized by whom and if ended up with a certificate

# Informal training in Hospital Management

Yes No

If yes, please specify title, date, organized by whom and if ended up with a certificate

# E-mail Address

| Training Needs Assessment for Public Hospital Managers | |  |
| --- | --- | --- |
| Hospital Characteristics | |  |
| 11. Mention Hospital characteristics as regarding TYPE of HOSPITAL | |  |
| District hospital | |  |
| Provincial hospital | |  |
| Regional Hospital | |  |
| Specialty hospital | |  |
| Other (please specify) | |  |
|  |  |  |
| 12. Mention Hospital characteristics as regarding TEACHING STATUS | |  |
| Teaching | |  |
| Non-teaching | |  |
| 13. Mention Hospital characteristics as regarding NUMBER of BEDS | |  |
| <100 | |  |
| 101-250 | |  |
| 251-400 | |  |
| >400 | |  |

| Training Needs Assessment for Public Hospital Managers |  |
| --- | --- |
| Please rate your level of need for training |  |
| 1. Please rate your level of need for training as regarding LEADERSHIP   No Need for Low Need for Moderate Need for High Need for Not Applicable to Training Training Training Training the Job  Leadership skills and behavior (e.g. vision, influence, decisive, energetic, flexible)  Leading and managing change  Other (please specify)   1. Please rate your level of need for training as regarding PERSONAL and INTERPERSONAL QUALITIES   No Need for Low Need for Moderate Need for High Need for Not Applicable to Training Training Training Training the Job  People interaction and relationship management  Communication skills 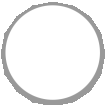 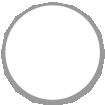 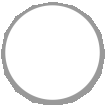 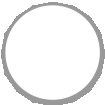 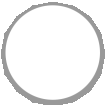 Facilitation, negotiation  and conflict resolution  Other (please specify)   1. Please rate your level of need for training as regarding BUSINESS SKILLS and KNOWLEDGE |  |


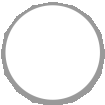

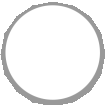

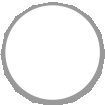

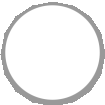

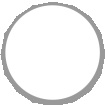

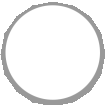


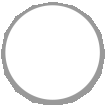

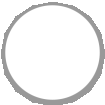

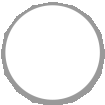

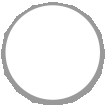

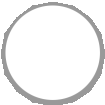

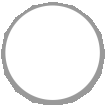

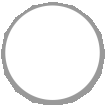

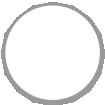

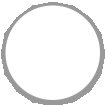

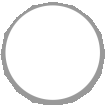

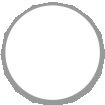

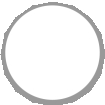

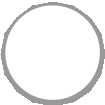

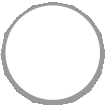


No Need for Training

Low Need for Training

Moderate Need for Training

High Need for Training

Not Applicable to the Job


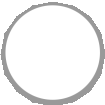

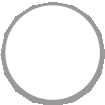

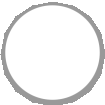

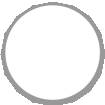

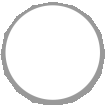


Strategic Planning

Systems thinking
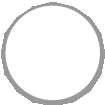

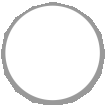

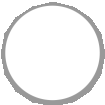

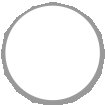

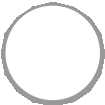


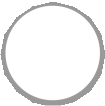

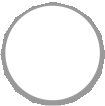

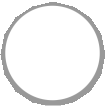

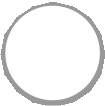

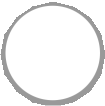


Evidence-informed decision making

Hospital Autonomy
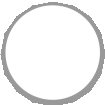

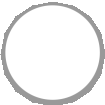

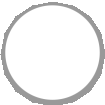

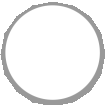

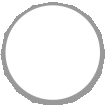


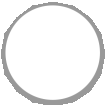

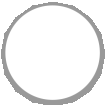

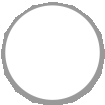

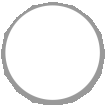

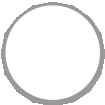


Problem solving and analysis


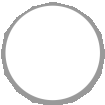

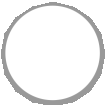

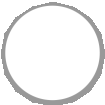

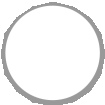

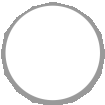
Coaching, mentoring and team building


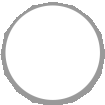

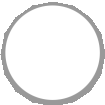

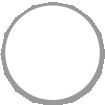

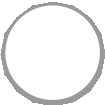

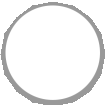


Knowledge of hospital dynamics

Rules and regulations
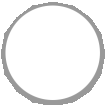

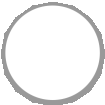

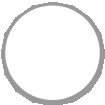

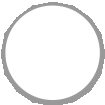

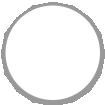


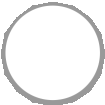

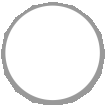

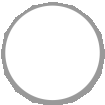

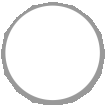

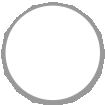


Human Resource Management


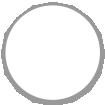

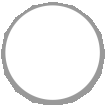

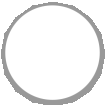

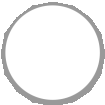

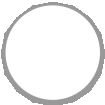
Managing Health Professionals


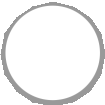

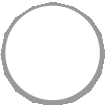

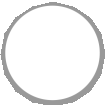

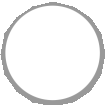

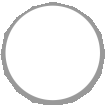


Delegating and assigning responsibility

Financial Management and resource allocation


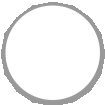

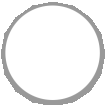

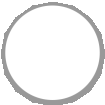

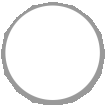

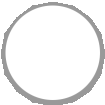


Hospital Efficiency


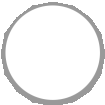

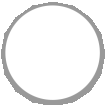

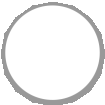

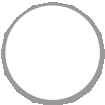

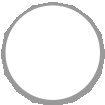

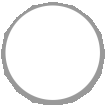

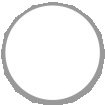

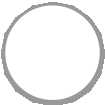

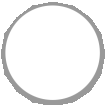

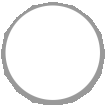
Contracting out
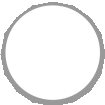

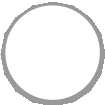

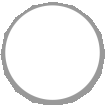

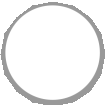

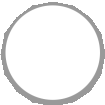


Operations, administration and resource management

Information Management

Quality Improvement

Performance

Measurement and Management

Risk Management

Knowledge Management

Supply Chain Management

Managing facilities and equipment

Program and Project Management

Monitoring and Evaluation

No Need for Training

Low Need for Training

Moderate Need for Training

High Need for Training

Not Applicable to the Job

Crisis Management

Disaster Management

Marketing

Computing Other (please specify)

# Please rate your level of need for training as regarding PROFESSIONAL and SOCIAL RESPONSIBILITY

No Need for Training

Low Need for Training

Moderate Need for Training

High Need for Training

Not Applicable to the Job

Personal and professional accountability

Community involvement

Response to patients and customers' needs

Contribution to profession

Ethical conduct

External relations and networking

Other (please specify)

# Please rate your level of need for training as regarding HEALTHCARE ENVIRONMENT

No Need for Training

Low Need for Training

Moderate Need for Training

High Need for Training

Not Applicable to the Job

Knowledge of health systems and organizations

Linking Hospital to Health Care System

Knowledge of public health

Knowledge of basic epidemiology

Knowledge of political environment

Other (please specify)

# What are the five most important Training needs you would identify?

1. What are the most important challenges that you face to manage your hospital?
